# Maternal and Fetal Outcomes in Women with Diabetes in Pregnancy Treated before and after the Introduction of a Standardized Multidisciplinary Management Protocol

**DOI:** 10.1155/2021/9959606

**Published:** 2021-11-12

**Authors:** Maddalena Morlando, Fabiana Savoia, Anna Conte, Antonio Schiattarella, Marco La Verde, Michela Petrizzo, Mauro Carpentieri, Carlo Capristo, Katherine Esposito, Nicola Colacurci

**Affiliations:** ^1^Prenatal Diagnosis and High-Risk Pregnancy Unit, Department of Woman, Child, and General and Specialised Surgery, University of Campania “Luigi Vanvitelli”, Naples, Italy; ^2^Unit of Diabetes, University of Campania “Luigi Vanvitelli”, Naples, Italy; ^3^Neonatal Intensive Care Unit, Department of Woman, Child, and General and Specialised Surgery, University of Campania “Luigi Vanvitelli”, Naples, Italy; ^4^Neonatal Care Unit, Department of Woman, Child, and General and Specialised Surgery, University of Campania “Luigi Vanvitelli”, Naples, Italy; ^5^Unit of Diabetes, Department of Medical and Surgical Sciences, University of Campania “Luigi Vanvitelli”, Naples, Italy

## Abstract

**Background:**

Diabetes in pregnancy is associated with an increased risk to the woman and to the developing fetus. Currently, there is no consensus on the optimal management strategies for the follow-up and the timing of delivery of pregnancies affected by gestational and pregestational diabetes, with different international guidelines suggesting different management options.

**Materials and Methods:**

We conducted a retrospective cohort study from January 2017 to January 2021, to compare maternal and neonatal outcomes of pregnancies complicated by gestational and pregestational diabetes, followed-up and delivered in a third level referral center before and after the introduction of a standardized multidisciplinary management protocol including diagnostic, screening, and management criteria.

**Results:**

Of the 131 women included, 55 were managed before the introduction of the multidisciplinary management protocol and included in group 1 (preprotocol), while 76 were managed according to the newly introduced multidisciplinary protocol and included in group 2 (after protocol). We observed an increase in the rates of vaginal delivery, rising from 32.7% to 64.5% (<0.001), and the rate of successful induction of labor improved from 28.6% to 86.2% (*P* < 0.001). No differences were found in neonatal outcomes, and the only significant difference was demonstrated for the rates of fetal macrosomia (20% versus 5.3%, *P*: 0.012). Therefore, the improvements observed in the maternal outcomes did not impact negatively on fetal and neonatal outcomes.

**Conclusion:**

The introduction of a standardized multidisciplinary management protocol led to an improvement in the rates of vaginal delivery and in the rate of successful induction of labor in our center. A strong cooperation between obstetricians, diabetologists, and neonatologists is crucial to obtain a successful outcome in women with diabetes in pregnancy.

## 1. Introduction

Diabetes in pregnancy is associated with an increased risk to the woman and to the developing fetus. Gestational diabetes mellitus (GDM) is characterized by glucose intolerance and insulin resistance recognized for the first-time during pregnancy. GDM is seen to be closely associated with adverse perinatal outcome and with an increased risk of developing type 2 diabetes mellitus (T2DM) in the future life for both the mother and the fetus [1]. It is estimated that approximately 10-15% of women who have diabetes during pregnancy have pregestational diabetes, either type 1 diabetes mellitus (T1DM) or T2DM [2]. Miscarriage, stillbirth, congenital malformations, preeclampsia, macrosomia, birth injury, perinatal mortality, neonatal hypoglycemia, and neonatal intensive care unit (NICU) admission are more common in women with preexisting diabetes [3]. Women with preexisting diabetes who are planning a pregnancy should be ideally managed by a multidisciplinary team including endocrinologist, diabetologist, maternal-fetal medicine specialist, and nutritionist when available [4].

There is currently no consensus regarding the optimal management strategies, the specific antepartum tests, the frequency of testing, and the timing of delivery of pregnancies affected by GDM and pregestational diabetes, with different international guidelines suggesting different management options. Induction of labor (IOL) is frequently suggested in order to reduce maternal and fetal adverse outcomes.

Diabetes in pregnancy is associated with an increased risk of stillbirth as pregnancy progresses [5–9]. Therefore, the increased neonatal morbidity and mortality associated with delivery before 39 weeks' gestation must be balanced with the increased risk of stillbirth with expectant management [10]. In addition, a policy of IOL at earlier gestational ages might be associated with a higher risk of failed induction and a rising risk of cesarean delivery (CD) [6–8]. Many strategies have been reported to identify women with at high risk of adverse outcomes, which might benefit more from a policy of earlier IOL [10–14]. The suggested gestational age for elective delivery varies between the different guidelines ranging from 36 to 39 for women with pregestational diabetes, whereas planned delivery from 38 to 40 is advised for women with GDM, further demonstrating that there is still lacking consensus to strongly recommend one gestational age over another [2, 15–19]. The aim of the present study is to compare maternal and neonatal outcomes of pregnancies complicated by GDM and pregestational diabetes, followed-up and delivered in a third level referral center before and after the introduction of a standardized multidisciplinary management protocol.

## 2. Materials and Methods

This is a retrospective cohort study conducted in a tertiary referral centre at the University of Campania “Luigi Vanvitelli.” Data were collected from the maternal and neonatal clinical notes of all women with a singleton pregnancy either with GDM or pregestational diabetes and attending our hospital for antenatal care between January 2017 and January 2021. Before July 2019, the management and the timing of delivery of women with diabetes were not codified in a common management protocol, and the decision about the frequency of the antenatal appointments, the timing, and the mode of delivery for each individual case was left to the discretion of the attending physician. In order to standardize the management process and to be consistent with the frequency and type of antenatal care provided to women with diabetes in pregnancy, on July 2019, a standardized multidisciplinary management protocol was written in close collaboration with the diabetologists and neonatologists involved in the antenatal management of women with diabetes and in the postnatal care of their infants. Since the publication and dissemination of the protocol among the hospital staff, all the obstetricians involved in the management of women with diabetes in pregnancy have strictly adhered to it. Women included in the present study were divided into two groups: the first group encompassing all women managed and delivered before the introduction of the multidisciplinary protocol (preprotocol group) and women managed according the multidisciplinary protocol (after protocol group). Maternal baseline characteristics and outcomes and fetal outcomes were compared among the two groups.

### 2.1. The Standardized Multidisciplinary Management Protocol

Our protocol is currently in use and it includes diagnostic and screening criteria as well as management criteria. The antenatal care, the timing and frequency of testing, and the timing of delivery are discussed and provided separately for women with pre-gestational and gestational diabetes (Table 1).

The diagnosis of GDM and pregestational diabetes is made in accordance to previously published criteria and in line with national guidelines [2, 19]. Women presenting at the first antenatal appointment with a fasting blood glucose ≥ 126 mg/dl (≥7.0 mmol/l) or a random blood glucose ≥ 200 mg/dl (≥11.1 mmol/l) or a hemoglobin A_1C_ ≥ 6.5% (48 mmol/mol) in two different nonconsecutive measurements before 12 weeks of gestation are classified as “overt diabetes in pregnancy” and therefore managed with the same criteria of pregestational diabetes.

Gestational diabetes is usually diagnosed following the screening performed with a one-step approach [20] using a 75 g, 2-hour oral glucose tolerance test (OGTT). The 75 g, 2-hour OGTT is offered at 16-18 weeks' gestation in women with a particularly high risk of GDM (prior GDM, first trimester fasting glucose 100-125 mg/dL, BMI ≥ 30 kg/m^2^), while it is performed at 24-28 weeks' gestation in the remaining women at risk (maternal age ≥ 35 years, BMI ≥ 25 kg/m^2^, prior fetal macrosomia, prior GDM with a negative screening at 16-18 weeks, first degree relative with T2DM, high risk ethnicity). GDM diagnosis is made when any single threshold value is met or exceeded (fasting value, 92 mg/dL; 1-hour value, 180 mg/dL; or 2-hour value, 153 mg/dL). The glycemic control during pregnancy was assessed in accordance to the American Diabetes Association criteria [21]. In women with continuous glucose monitoring and T1DM, the glycemic control was considered good if >70% of readings per day were within target glucose range of 63–140 mg/dL, if <4% of readings per day were below target glucose range, and if <25% of readings per day were above target glucose range. In women with continuous glucose monitoring and T2DM or GDM, the glycemic control was considered good if >90% of readings per day were within target glucose range of 63–140 mg/dL, if <5% of readings per day were below target glucose range, and if <5% of readings per day were above target glucose range. In women without continuous glucose monitoring, the same percentages of readings per day within target glucose range were roughly applied, on the basis of 3 to 10 readings per day.

#### 2.1.1. Gestational Diabetes

Antenatal care of women with GDM is provided in the high-risk pregnancy antenatal clinic. In addition to the routine pregnancy care and assessment, an intensive counseling regarding the complications of pregnancy associated with GDM is provided. Fetal growth and amniotic fluid volume are evaluated at each antenatal appointment. The woman is referred to a dedicated team of diabetologists highly experienced in the management of pregnant women. A dietary plan tailored on the individual woman BMI, habits, and needs is provided by a nutritionist, and mild to moderate physical activity is encouraged. The need for maternal cardiac assessment is evaluated in conjunction with the diabetologists in order to identify women at increased risk of hypertensive disorders of pregnancy. The following antenatal appointments are usually scheduled every 4 weeks, both for obstetrics and diabetologists' reevaluation. Antenatal fetal monitoring with weekly cardiotocography is planned from 36 weeks of gestation. Women are informed on how to monitor glycemia at home which is usually advised from 3 to 10 times per day. The glycemic goals to be achieved during pregnancy, if compatible with adequate fetal growth and with no episodes of hypoglycemia, are

- <90 mg/dl fasting glucose
- <130 mg/dl 1 hour after meal
- <120 mg/dl 2 hours after meal
- *Hemoglobin* *A*_1*c*_ < 6.5%

#### 2.1.2. Delivery Criteria in GDM Women

The delivery criteria applied in the multidisciplinary management protocol take into account both the metabolic control by maternal blood glucose levels (defined by the diabetologists) and two fetal characteristics which have been associated with fetal hyperglycemia: the amniotic fluid volume [6, 16] and the fetal growth centile [22]. The rationale for the inclusion of these criteria in the definition of the optimal time of delivery is based on the fact that in some women; despite the evidence of optimal glycemic values at 3 to 10 daily measurements, a certain degree of hyperglycemia can still be present, with an impact on fetal growth and amniotic fluid production which can be detected at ultrasound assessment. We offer a close monitoring of fetuses presenting with increased amniotic fluid and/or increased growth, which might be at increased risk of adverse outcome [23].

Therefore, in women with a good glycemic control, delivery is planned according to the following criteria:

- If estimated fetal weight is <97^th^ centile and amniotic fluid volume is normal (either with a deepest pocket between 2 and 8 cm, or with an *amniotic* *fluid* *index* < 24 *cm*), admission is scheduled at 39^+0^ weeks, and IOL or CD is planned at 39^+1^ weeks
- If estimated fetal weight is ≥97^th^ centile and/or the amniotic fluid is increased (either with a *deepest* *pocket* ≥ 8 *cm* or with an *amniotic* *fluid* *index* ≥ 24 *cm*, *maximum* *pocket* > 8 *cm*, *AFI* < 24), admission is scheduled at 38^+0^ weeks for daily monitoring of fetal well-being. If there are no concerns about fetal well-being, IOL or CD is planned at 39^+1^ weeks

If the woman has no optimal glycemic control despite insulin therapy, admission is scheduled from 37^+1^ weeks for daily monitoring of fetal well-being, and delivery is planned within 38^+0^ weeks.

At earlier gestations, in women with poor glycemic control despite insulin treatment, hospitalization can be offered in an attempt to safely and aggressively optimize glucose control by improving the dietary compliance and by accurate monitoring of blood glucose levels. A conservative management is usually undertaken until 34^+1^ weeks. After this time, delivery can be considered as the safest mode of management if glycemic control is poor despite insulin therapy and despite admission.

IOL is usually performed with a vaginal prostaglandin pessary (dinoprostone 10 mg). If there is no onset of labor, the vaginal pessary is left in situ for 24 hours. A new pessary is inserted after a 24-hour break. A maximum of three attempts is allowed. At last, feasibility of oxytocin infusion and/or amniorexis is evaluated. Failed induction is diagnosed when there is either no possibility to proceed with oxytocin infusion and/or amniorexis (e.g., unfavorable cervix with a Bishop score < 4) or no cervical changes despite at least 8 hours of oxytocin infusion and regular uterine contractions. In case of failed induction, a CD is performed.

#### 2.1.3. Pregestational Diabetes

At the first antenatal appointment in the high-risk pregnancy clinic, extensive counseling regarding the risks and complications of pregnancy associated with diabetes is performed. Given the evidence of an increased risk of congenital abnormalities, especially anencephaly, microcephaly, and congenital heart disease, directly proportional to hemoglobin A_1C_ during the first 10 weeks of pregnancy, a strict glycemic control is strongly encouraged [24]. Usually, the diabetologists are already informed about the pregnancy, and a close contact with them is ensured in order to plan the following examinations:

- Baseline evaluation of TSH, microalbuminuria, and electrocardiogram
- Baseline evaluation by ophthalmologist, dietitian, cardiologist, or nephrologist
- Regular ongoing assessment of blood glucose values and hemoglobin A_1c_
- Ketonemia/ketonuria in case of intercurrent infections/conditions

Women with T1DM and T2DM are usually already informed on how to monitor glycemia at home. Women with pregestational diabetes are usually prescribed folic acid 5 mg/daily in the first trimester in order to reduce the risk of neural tube defects [17, 25] and low-dose aspirin 100–150 mg/day from the end of the first trimester until 34 weeks' gestation, in order to lower the risk of preeclampsia [26]. A detailed ultrasound anatomical survey is carried out at 16-18 weeks and again at 20-22 weeks. Fetal echocardiography is performed at 24-26 weeks. Fetal growth and amniotic fluid volume are evaluated at each antenatal appointment, which are scheduled monthly until 28 weeks and every 3 weeks afterwards. Antenatal fetal monitoring with weekly cardiotocography is planned from 34 weeks of gestation. The glycemic goals to be achieved during pregnancy are the same reported above for the women with GDM.

#### 2.1.4. Delivery Criteria in Pregestational Diabetes

In women with pregestational diabetes with a good glycemic control, delivery is planned according to the following criteria:

- If estimated fetal weight is <97th centile and amniotic fluid volume is normal (either with a deepest pocket between 2 and 8 cm or with an *amniotic* *fluid* *index* < 24 *cm*), admission is scheduled at 37^+6^ weeks, and IOL or CD is planned from 39^+0^ weeks. Delivery must take place within 40^+1^weeks
- If estimated fetal weight is ≥97th centile and/or the amniotic fluid is increased (either with a *deepest* *pocket* ≥ 8 *cm* or with an *amniotic* *fluid* *index* ≥ 24 *cm*, *maximum* *pocket* > 8 *cm*, *AFI* < 24), admission is scheduled at 36^+0^ weeks for daily monitoring of fetal well-being. If there are no concerns about fetal well-being, IOL or CD is planned from 37^+2^ weeks. Delivery must take place within 38^+4^ weeks

If the woman has no optimal glycemic control despite increase in the insulin therapy, admission can be considered to optimize glucose control and for close monitoring of fetal well-being, and delivery is planned within 38^+0^ weeks. A conservative management is usually undertaken until 34^+1^ weeks. After this time, delivery can be considered if glycemic control is poor despite increase in insulin therapy and despite admission.

### 2.2. Main Outcome Measures

The maternal baseline antenatal characteristics compared between the two groups of women included in the present study were age, BMI, type of diabetes (pregestational or GDM), and the number of previous vaginal deliveries. The following maternal outcome measures were compared between the 2 groups of women that included the gestational age at the time of delivery, the rates of women undergoing IOL, the rate of response to IOL, the mode of delivery (either vaginal or CD), the rate of operative vaginal delivery, the need for episiotomy, the occurrence of perineal tears, the occurrence of postpartum hemorrhage (PPH), and the length of the first and of the second stage of labor.

The following neonatal outcomes were also compared: the birthweight, the Apgar score at 1 and 5 minutes, the umbilical cord pH, the occurrence of macrosomia (defined as birthweight >4000 gr) and shoulder dystocia, the rates of NICU admission, the length of NICU stay, and the rates of respiratory distress syndrome, sepsis, and asphyxia. We also evaluated the need for hypothermia, the occurrence and length of hypoglycemia, and the need for any kind of respiratory support.

#### 2.2.1. Statistical Analyses

Statistical analysis was performed using Statistical Package for Social Sciences (SPSS) v. 20.0 (IBM Inc., Armonk, NY, USA). Data were shown as means ± standard deviation or number (percentage). Chi-square test was performed for categorical variables. Student's *t*-test was used for comparison of means values of the two groups for continuous variables. Mann–Whitney test was used for nonparametric variables. All the analyses were performed using a two-sided model, considering a normal distribution as appropriate. *P* value less than 0.05 was considered statistically significant.

## 3. Results

During the four-year study period, 133 women with a singleton pregnancy with gestational or pregestational diabetes referred to our institution were managed and delivered at our referral center. Two women with multiple pregnancies (one twin pregnancy and one triplet pregnancy) were excluded from the present study. Therefore, among the 131 women left, 55 were managed before the introduction of the multidisciplinary management protocol and were therefore included in group 1 (preprotocol), while 76 were managed according to the newly introduced multidisciplinary protocol and were therefore included in group 2 (after protocol).

Baseline maternal antenatal characteristics of the women included into the two groups are presented in Table 2. There were no significant differences between the two groups. Importantly, there were no differences in the proportion of women with pregestational and gestational diabetes included into the two groups (*P*: 0.225), allowing the comparison among them.

Maternal outcomes of women included are shown in Table 3. The mean of gestational age at delivery did not differ between the groups. Despite not significant, the rates of women undergoing IOL showed a trend of increase in the group 2 (25.5% versus 38.2%, *P*: 0.137). Interestingly, the only maternal outcomes showing a significant difference between the 2 study periods were the mode of delivery and the response to IOL. Indeed, after introduction of the multidisciplinary protocol, we observed an increase in the rates of vaginal delivery, rising from 32.7% to 64.5% (<0.001). In addition, among women undergoing IOL, the rates of women experiencing a successful vaginal delivery rose from 28.6% to 86.2% (*P* < 0.001).

Neonatal outcomes are shown in Table 4. Data were missing for nine infants of group 2; therefore, the overall number of infants included in this group (*n*: 67) is different from the number of women included in the same group (*n*: 76). There were no differences in the groups (*P*: 0.214). Despite an apparent improvement in several neonatal outcome measures following the introduction of the management protocol, the only significant difference was demonstrated for the rates of fetal macrosomia (20% versus 5.3%, *P*: 0.012). The occurrence of fetal hypoglycemia showed a reduction trend in the group 2 (26.8% versus 14.9%, *P*: 0.119); however, this difference was not significant.

## 4. Discussion

In this retrospective cohort study, we compared maternal and neonatal outcomes of pregnancies complicated by GDM and pregestational diabetes, delivered at out referral center before and after the introduction of a standardized multidisciplinary management protocol. The main finding of the present study is the significant improvement observed in some of the maternal outcomes after the introduction of the management protocol. Above all, the rates of vaginal delivery rose from 32.7% to 64.5% (<0.001), and the rate of successful IOL improved from 28.6% to 86.2% (*P* < 0.001). At the same time, we found no differences in fetal and neonatal outcomes, apart from a significant reduction in the occurrence of fetal macrosomia (20% versus 5.3%, *P*: 0.012). This is an important finding, proving that the improvements observed in the maternal outcomes did not impact negatively on fetal and neonatal outcomes. This may also suggest that after the introduction of the standardized management protocol, the selection of women undergoing IOL and the choice of the timing of IOL were performed in a more effective way, leading to a reduction in the CS rate and a better response to IOL. This may also suggest that building a multidisciplinary team and ensuring a strong cooperation and interaction between the obstetricians, the diabetologists, and the neonatologists is crucial to obtain a successful outcome in women with diabetes in pregnancy.

One key factor in the management of pregnancies complicated by diabetes is determining at what point in gestation the risk of expectant management outweigh the risk of delivery. Diabetes in pregnancy is associated with and increased risk of stillbirth as pregnancy progresses [16]. Although the risk for stillbirth is particularly increased when glycemic control is poor, this risk is still higher than the general population especially in women with pregestational diabetes even when there is adequate glycemic control [27]. As a result, several attempts have been tried to identify women with a particularly high risk of adverse outcomes, which might benefit more from an intensification of antenatal surveillance or a policy of earlier IOL [10–14].

In view of this, in our multidisciplinary management protocol, we opted to consider as women at higher risk of pregnancy complication not only the ones with poor glycemic control but also the ones with adequate glycemic control showing an increase in the amniotic fluid volume [6, 16] and/or an excessive fetal growth (>97^th^ centile) [22].

One possible explanation to the evidence of excessive fetal growth even in the presence of a good glycemic control is that limited episodes of hyperglycemia have been demonstrated to have similar effects as prolonged hyperglycemia in upregulating glucose and amino acid intake [28].

An additional explanation is provided by a recent study showing a higher risk of delivering an infant large for gestational age (LGA) in women with a poor glycemic control during the first trimester, while glycemic control in later trimesters did not affect this risk [29]. Indeed, the placenta is a vital organ supporting fetal development and ensuring the transport of nutrients to the fetus. It also acts as an endocrine organ, releasing hormones to promote placental and fetal growth and also influencing maternal metabolism [30]. Placental development occurs during the first trimester; therefore, uncontrolled glycemia during this period might interfere with optimal placental development, and this may explain why the neonatal birthweight has been proven to be mostly affected by glycemic control than in the first trimester of pregnancy.

In comparison with current guidelines our protocol suggests, for women with GDM, elective delivery at 39^+1^ weeks when metabolic control is good. Delivery is planned between 37^+0^ and 38^+0^ weeks in women with no optimal glycemic control despite insulin therapy. In women with pregestational diabetes with a good glycemic control, delivery is advised from 37^+2^ to 39^+0^ weeks. If the woman has no optimal glycemic control despite increase in the insulin therapy, delivery must occur within 38^+0^ weeks. For both women with GDM and pregestational diabetes at earlier gestations, delivery is advised only on an individual basis in cases with a particular high risk of adverse outcome (e.g., fetal growth restriction, preeclampsia, and diabetic complications). The literature and the guidelines regarding timing of delivery of women with diabetes in pregnancy are quite heterogeneous, and there have been few quality studies to assess the optimal management for these patients.

NICE guidelines [2] advise women with GDM to give birth no later than 40^+6^ weeks. While women with type 1 or type 2 diabetes, no other complications are advised to have an elective birth by between 37 weeks and 38^+6^ weeks. The American College of Obstetricians and Gynecologists suggest delivery of women with GDM at 38-39 weeks, while for women with pregestational diabetes early delivery between 36^+0^ to 38^+6^ is indicated in women with particularly high risk. In contrast, women with well-controlled diabetes can be managed expectantly to until 39^+6^ weeks of gestation.

The Canadian Diabetes Association [15] recommends pregnant women with either gestational or pregestational diabetes should be offered induction between 38 to 40 weeks' gestation depending on their glycemic control and other comorbidity factors. The recommendations of the main international societies involved in the care of women with diabetes in pregnancy are summarized in Table 5.

Previous studies have investigated the risks and benefits of elective delivery versus expectant management in women with diabetes. The only randomized controlled trial on induction of labor versus expectant management in women with GDM between 38^+0^ and 39^+0^ weeks found no differences between the 2 groups [31]. However, due to difficulties in the recruitment, the study was ended without achieving the planned sample size. A retrospective study including 193.028 deliveries to women with GDM [32] found that when the risk of planned delivery (as quantified by the risk of infant death at a given gestational age) is compared with the risk of expectant management for one week in women with GDM, the risk of delivery is higher than expectant management at 36 weeks, while at 39 weeks, the risk of expectant management exceeds that of delivery (RR 1.8, 95% CI: 1.2–2.6). Given that neonatal morbidity did not appear to be higher at 39 weeks as compared with 40 weeks, the authors suggested that 39 weeks may be the best timing at which to plan delivery in order to decrease infant mortality.

There is insufficient evidence on timing of delivery for women with pregestational diabetes. A recent population-based study [33] further supported delivery at 38, 39, or 40 weeks of gestation for women with diabetes. The authors found no maternal benefit and little or no additional neonatal benefit for scheduled delivery at 39 rather than 38 weeks of gestation for women with type 1 or type 2 diabetes. Therefore, as these women have a much greater risk of stillbirth compared with women with GDM and given that a strict glycemic control is challenging to achieve in women with type 1 diabetes, the authors' conclusion is that there is little justification for delaying delivery of women with preexisting diabetes beyond 38 weeks of gestation.

Given the current guidelines, as well as the available evidence, it seems reasonable to consider delivery at 39 weeks' gestation, even in relatively well-controlled women with GDM. This was also the rationale of our management protocol. We reckon that the optimal time of delivery in these women is still matter of debate, and future randomized controlled studies should be conducted to examine this clinical intervention.

IOL is commonly thought to be associated with and increased risk of CD. However, in a Cochrane meta-analysis of randomized trials, women who were induced were actually proven to have a lower risk of CD [34]. In a specific population of diabetic women, the only randomized controlled trial of induction of labor versus expectant management demonstrated that women induced at 38 weeks' gestation as compared with expectant management had no difference in the rate of CD. In addition, in the expectant management group, there was an increased prevalence of LGA infants (23% vs. 10%) and shoulder dystocia (3% vs. 0%). The available evidence suggests that induction of labor in women with diabetes does not increase the risk of CD. This is particularly true when adequate selection of women is performed. Our findings are in line with the reported evidence. In fact, after the introduction of the multidisciplinary protocol allowing an accurate selection of women for IOL, we observed a huge reduction in the CD rate, which become similar to the total CD rate at our institution (34% in 2019 and 2020). We reckon that all women underwent IOL by vaginal dinoprostone 10 mg and no other methods for IOL were used in our cohort. Therefore, our results may not apply perfectly to different centers with different induction protocols.

The main strength of the present study is the presence of a well-codified and reproducible management protocol, which was strictly applied after its introduction. This is proven by the fact that all the women managed after the introduction of the protocol were delivered according to indications that matched the protocol criteria. Forty-seven women did not undergo IOL due to several reasons. Twenty-four women gave birth spontaneously before the planned time for delivery, and among them, three had a preterm delivery. Twenty-three women underwent a planned CD due to different indications: 13 women because of history of ≥1 prior CDs; 8 women had a fetal or maternal indication (3 for maternal rethinopathy, 2 for fetal growth restriction, 1 for macrosomia, 1 for breech presentation, 1 for poorly controlled diabetes at 34 weeks). Among women undergoing spontaneous labor before the planned time for delivery, two women underwent emergency CD: one for abnormal cardiotocography and one for failure to progress in labor. The women included in the two study groups were homogeneous in terms of their baseline characteristics, in particular, in terms of women affected by pregestational diabetes. We therefore speculate that the improvements seen in maternal and neonatal outcomes were actually due to the introduction of the protocol and to the improvements in the cooperation and interaction between the physicians involved in the multidisciplinary team. One more strength is the fact that we analyzed both maternal and infant complications. This is particularly important, as often, in obstetric decision-making benefits for the infant may increase the chance of harm to the mother, and vice versa [33].

The main limitations of this study are the retrospective nature and the limited sample size, which may have limited the strength of our results. However, the differences in the main maternal outcomes were wide, and they reached statistical significance despite the relatively small numbers. Given the retrospective design in our study, we lacked data on important confounders, like the glycemic control in the first trimester and the hemoglobin A_1c_ values, which are critical to define the level of risk for women with pregestational diabetes. The lower rates of macrosomia in the group of women who delivered after introduction of the study protocol might indicate a higher rate of well compensated women. We can speculate that this was due to the improvements related to the introduction of the study protocol but we cannot rule out a possible higher prevalence of women with poorer glycemic control in the first group. Similarly, we lack data on which diagnostic criteria were used to diagnosis GDM in the first group of women. However, the criteria for the diagnosis of GDM have been included in the national guidelines for the management of low-risk pregnancy in 2011 [35], and we therefore assume that the same criteria were applied also before the introduction of our multidisciplinary protocol. One additional limitation of the present study is the lack of data on the gestational age at diagnosis of GDM. Italian guidelines suggest screening for gestational diabetes at 16–18 or 24–28 weeks of gestation (or both) depending on the personal risk profile. The initial acceleration of fetal growth and fat mass accretion in GDM mothers were demonstrated to be already detectable at 20 weeks of gestation [36]. In addition, women diagnosed with GDM at 16–18 weeks of gestation have been proven to deliver infants with a lower birthweight compared with neonates born to women diagnosed at 24–28 weeks of gestation [37], most likely due to an early and adequate treatment of hyperglycemia. Therefore, the gestational age at the diagnosis and at the initial treatment might have influenced the rates of macrosomia in the two groups.

## 5. Conclusion

The introduction of a standardized multidisciplinary management protocol led to an improvement in the rates of vaginal delivery and in the rate of successful IOL in our referral centre. At the same time, we found no differences in fetal and neonatal outcomes, apart from a significant reduction in the occurrence of fetal macrosomia. These findings are showing that the improvements observed in the maternal outcomes did not impact negatively on fetal and neonatal outcomes. Our findings demonstrate that building a multidisciplinary team and ensuring a strong cooperation and interaction between the obstetricians, the diabetologists, and the neonatologists is crucial to obtain a successful outcome in women with diabetes in pregnancy. The optimal management strategies and the optimal time of delivery of women with diabetes in pregnancy are still debated. Future randomized trials will have to focus on these important research questions.

## Figures and Tables

**Table 1:** The standardized multidisciplinary management protocol details.

	Antenatal care	Delivery criteria
Pregestational diabetes	(i) Counseling regarding the risks and complications associated with diabetes in pregnancy (ii) Baseline evaluation of thyroid function, microalbuminuria, electrocardiogram, and baseline evaluation by ophthalmologist, dietitian, cardiologist, and nephrologist (iii) Regular assessment of blood glucose values and hemoglobin A1c (iv) Ketonemia/ketonuria in case of intercurrent infections/conditions (v) Detailed ultrasound anatomical survey at 16-18 weeks and at 20-22 weeks (vi)Fetal echocardiography at 24-26 weeks (vii) Antenatal appointments (with assessment of fetal growth and amniotic fluid volume) are scheduled monthly until 28 weeks and every 3 weeks afterwards (viii) Weekly cardiotocography is planned from 34 weeks of gestation	In women with a good glycemic control
		(a) If EFW < 97th centile and AFV is normal, admission at 37^+6^ weeks and IOL or CD is planned from 39^+0^ weeks. Delivery must take place within 40^+1^weeks (b) If EFW ≥ 97^th^ centile and/or AFV is increased, admission at 36^+0^ weeks for daily monitoring of fetal well-being If there are no concerns about fetal well-being IOL or CD is planned from 37^+2^ weeks, delivery must take place within 38^+4^ weeks.
		In women with no optimal glycemic control despite increase in the insulin therapy
		Admission can be considered to optimize glucose control and for close monitoring of fetal well-being, and delivery is planned within 38^+0^ weeks. A conservative management is usually undertaken until 34^+1^ weeks.

Gestational diabetes	(i) Counseling regarding the risks and complications associated with diabetes in pregnancy (ii) Woman is referred to a team of highly experienced diabetologists, and a dietary plan is provided by a nutritionist. Physical activity is encouraged (iii) Antenatal appointments (with fetal growth and amniotic fluid volume) are scheduled monthly, both for obstetrics and diabetologists reevaluation (iv) Cardiac assessment is evaluated in conjunction with the diabetologists to identify women at increased risk of hypertensive disorders (v) Weekly cardiotocography is planned from 36 weeks of gestation (vi) Women are informed on how to monitor glycemia at home which is usually advised from 3 to 10 times per day	In women with a good glycemic control
		(a) If EFW is <97^th^ centile and AFV is normal, admission is scheduled at 39^+0^ weeks and IOL or CD is planned at 39^+1^ weeks (b) If EFW is ≥97th centile and/or the AFV is increased, admission is scheduled at 38^+0^ weeks for daily monitoring of fetal well-being If there are no concerns about fetal well-being IOL or CD is planned at 39^+1^ weeks.
		In women with no optimal glycemic control despite insulin therapy
		Admission is scheduled from 37^+1^ weeks for daily monitoring of fetal well-being, and delivery is planned within 38^+0^ weeks. At earlier gestations, in women with poor glycemic control, hospitalization can be offered to optimize glucose control by improving the dietary compliance and by accurate monitoring of blood glucose levels. A conservative management is usually undertaken until 34^+1^ weeks.

IOL: induction of labor; CD: cesarean delivery; EFW: estimated fetal weight; AFV: amniotic fluid volume.

**Table 2:** Maternal antenatal characteristics of women with diabetes in pregnancy who delivered before (group 1) and after (group 2) the introduction of a standardized multidisciplinary management protocol.

	Group 1	Group 2	*P*	
	Before protocol	After protocol		
	*n*: 55	*n*: 76		
Number of prior vaginal deliveries	0.4 ± 0.7	0.7 ± 1.2	0.113	
Maternal age, years	33.6 ± 5.3	34.1 ± 4.7	0.558	
BMI, kg/m^2^	29.9 ± 7.9	30.5 ± 45.3	0.633	
Type of diabetes			0.225	
Gestational diabetes	44 (80)	67 (88.2)		
Pregestational diabetes	11 (20)	9 (11.8)		

BMI: body mass index. Data are given as number (percentage) or mean ± standard deviation.

**Table 3:** Maternal outcomes of women with diabetes in pregnancy who delivered before (group 1) and after (group 2) the introduction of a standardized multidisciplinary management protocol.

	Group 1	Group 2	*P*
	Before protocol	After protocol	
	*n*: 55	*n*: 76	
Gestational age at delivery, weeks	38.2 ± 1.5	38.5 ± 2.3	0.476
Induction of labor	14 (25.5)	29 (38.2)	0.137
Response to induction^∗^	**4 (28.6)**	**25 (86.2)**	**<0.001**
Mode of delivery			**<0.001**
Vaginal delivery	**18 (32.7)**	**49 (64.5)**	
Cesarean section	**37 (67.3)**	**27 (35.5)**	
Operative vaginal delivery^∗∗^	4 (22.2)	6 (12.2)	0.439
Episiotomy^∗∗^	8 (44.4)	11 (22.4)	0.124
Vaginoperineal tears^∗∗^	8 (44.4)	28 (57.1)	0.439
Postpartum hemorrhage	3 (5.5)	1 (1.3)	0.309
Length of first stage, minutes	164.2 ± 120.9	155.1 ± 140.2	0.799
Length of second stage, minutes	48.6 ± 40.1	44.4 ± 38.6	0.688

Data are given as number (percentage) or mean ± standard deviation. Significant values in bold. ^∗^These numbers and percentages refer to women undergoing induction of labor. ^∗∗^These number and percentages refer to women with vaginal delivery.

**Table 4:** Neonatal outcomes of infants delivered by women with diabetes in pregnancy who delivered before (group 1) and after (group 2) the introduction of a standardized multidisciplinary management protocol.

	Group 1	Group 2	*P*
	Before protocol	After protocol	
	n: 55	n: 67	
			
Birthweight, grams	3453.4 ± 813.3	3311 ± 487.9	0.214
1 min. Apgar score	7.4 ± 1.7	7.6 ± 1.9	0.537
5 min. Apgar score	8.9 ± 1	9.1 ± 0.9	0.110
Umbilical cord pH	7.3 ± 1.1	7.3 ± 1.2	0.105
Fetal macrosomia	**11 (20)**	**4 (5.3)**	**0.012**
Shoulder dystocia^∗^	3 (16.7)	1 (2)	0.056
NICU admission	21 (38.2)	17 (25.4)	0.173
Length of NICU stay (days)	9.5 ± 7	8 ± 12.1	0.642
Respiratory distress syndrome	10 (18.2)	7 (10.4)	0.297
Sepsis	7 (12.7)	3 (4.5)	0.183
Asphyxia	4 (7.3)	5 (7.5)	1.000
Hypothermia	2 (3.6)	2 (3)	1.000
Hypoglycemia	15 (27.3)	10 (14.9)	0.119
Length of hypoglycemia (days)	1.3 ± 0.62	1.1 ± 0.32	0.284
Need for respiratory support	9 (16.4)	9 (13.4)	0.799

NICU: neonatal intensive care unit admission. Data are given as number (percentage) or mean ± standard deviation. Data were missing for 9 infants of group 2; therefore, the overall number of infants included in this group (*n*: 67) is different from the number of women included in the same group (*n*: 76). ^∗^These number and percentages refer to women with vaginal delivery (group 1: 18 women–group 2: 49 women). Significant values in bold.

**Table 5:** Comparison of different international guidelines regarding the optimal time of delivery in women with diabetes in pregnancy. GDM: gestational diabetes mellitus.

Authority	Recommendation
National Institute for Health and Clinical Excellence (2015) [2]	Advise pregnant women with type 1 or type 2 diabetes and no other complications to have an elective birth by induced labor or (if indicated) caesarean section, between 37 weeks and 38 weeks plus 6 days of pregnancy.
	Consider elective birth before 37 weeks for women with type 1 or type 2 diabetes who have metabolic or other maternal or fetal complications.
	Advise women with gestational diabetes to give birth no later than 40 weeks plus 6 days. Offer elective birth by induced labor or (if indicated) by caesarean section to women who have not given birth by this time.
	Consider elective birth before 40 weeks plus 6 days for women with gestational diabetes who have maternal or fetal complications.

Canadian Diabetes Association (2019) [15]	Pregnant women with either gestational or pre-gestational diabetes should be offered induction between 38 to 40 weeks gestation depending on their glycemic control and other comorbidity factors.
	In the view that the risk of intrauterine fetal death appears to outweigh the risk of infant death after 39 weeks, induction of labor at 39 weeks could be considered in insulin-treated GDM patients.
	In women with diet-controlled GDM induction by 40 weeks may be beneficial.

American College of Obstetricians and Gynecologists (2018) [16, 17]	Delivery of women with GDM at 38 weeks or 39 weeks of gestation would reduce overall perinatal mortality without increasing cesarean delivery rates.
	For women with pregestational diabetes early delivery (36 0/7 weeks to 38 6/7 weeks of gestation, or even earlier) may be indicated in some patients with vasculopathy, nephropathy, poor glucose control, or a prior stillbirth.
	In contrast, women with well-controlled diabetes with no other comorbidities may be managed expectantly to 39 0/7 weeks to 39 6/7 weeks of gestation as long as antenatal testing remains reassuring.
	Expectant management beyond 40 0/7 weeks of gestation generally is not recommended.

The Royal Australian and New Zealand College of Obstetricians and Gynaecologists (2021) [18]	If well managed with medical nutrition therapy and no fetal macrosomia or other complications, wait for spontaneous labor (unless there are other indications for induction of labor).
	If suspected fetal macrosomia or other complications, consider birth from 38^+0^ to 39^+0^ weeks' gestation.
	Suspected fetal macrosomia alone is not an indication for induction of labor before 39^+0^ weeks' gestation.
	In most cases, women with optimal blood glucose levels who are receiving pharmacological therapy do not require expedited birth before 39^+0^ weeks gestation.

The Australasian Diabetes in Pregnancy Society (2019) [19]	Women with preexisting diabetes should be advised to give birth by the end of 38 completed weeks' gestation, depending on the presence of fetal macrosomia, glycemic levels and any other complicating factors.

## Data Availability

All the data used in the present study can be accessed upon request to the corresponding author.
